# Some Human Lessons in War-Time

**Published:** 1916-10-07

**Authors:** 


### LIFE AND DEATH.

### Some Human Lessons in War-Time.

### III. CHILDHOOD AND THE CHILDREN.*

A cultured mind in the parent is of vital importance to the child; of what importance is seen in Wordsworth's words "the child is father to the man." But Wordsworth's words might be taken to mean, "No matter what you do for a child, what you teach it, how you try to influence it, you will not change its nature; as is the child, so will be the fully-developed man." And judging from the disregard of child-life so widely shown in these days, it might well be supposed that this was the feeling of the nation at large. For experience in public-health work and in the life and surroundings of the poor does, unfortunately, provide us with terrible facts relating to the neglect and environment of the children. It is indisputable that the greatest asset a nation has is its children, yet the life of a child, even in a Christian country, is ill-tended and not adequately safeguarded. Indeed, so little is it valued that the medical officer of health must often be impressed with the fact, in proportion to his sympathy with children, when he has to deal with the avoidable miseries and surroundings which contribute to the undoing and misery of childhood. Small wonder if he feels that it would be better for thousands of such children had they never been born. It is to be hoped this terrible condition of affairs will speedily disappear after the triumph of the Allies and the restoration of peace. Otherwise the present awakening apprehension of the individual to his spiritual nature amongst people of all classes, and the restoration to our fighting men of the vision which a former generation had, as we have pointed out already, will vanish with the declaration of peace, a contingency which no great statesman, nor any Christian man or woman, will desire.

### The Teaching of Christ.

In this series of papers dealing with death and life, with some human lessons in war-time, we desire to secure an earnest response to the call to individuals, and the nation at large, to realise their spiritual nature, for with it should come the quickening of the senses to the meaning of Christ's teaching: "Except ye be converted and become as little children, ye shall not enter into the kingdom of heaven. Whosoever, therefore, shall humble himself as this little child, the same is greatest in the kingdom of heaven. And whoso shall receive one such little child in My name receiveth Me." The lesson in regard to health and life which these words of Christ convey, like the vision of our forbears, has been largely lost, and so far as reality is concerned, with the majority probably, had almost become a dead letter before war was declared. The underlying causes of this we propose to demonstrate in large measure when dealing with money, its limitations, its impotence and its cure. And yet the child is part of the trusteeship which every human parent inherits for his or her own life. We cannot put this point clearer than in Carlyle's words, always incisive and always to the point. "Good Christian people, here lies for you an inestimable loan—the child; take all heed thereof, in all carefulness employ it; with high recompense, or else with heavy penalty will it one day be required back."

### Birmingham's Example: 1870.

We are fully aware that the renewal of the great fight for Bible teaching in public elementary schools in comparatively recent years may be accepted as evidence on this question of the mind of large numbers of the population. The present writer has the grateful remembrance of being closely associated with the victory over the Caucus in Birmingham in 1870, when Bible reading was carried there at the first School Board election. Bible reading has been continued in the Birmingham schools ever since, and we do not suppose there is a single honest man or woman in that great city to-day, who realises his or her own spiritual nature, who is not grateful for the victory won in 1870. How important definite religious instruction based upon the Bible and Christ's teaching is to a Christian nation we shall endeavour to explain directly. But first let us drive home an appalling anomaly—the fact that in this matter of the child-life of the nation, whereas there properly has been for nearly fifty years a strenuous fight to give the mind of the children of the nation free access to God's Word through the teacher, that is to say, to recognise each infant's spiritual nature, the physical requirements and claims of the child have been criminally neglected until comparatively recent years. They are even still wholly inadequate, as shown by the high mortality amongst the children of the nation, and especially amongst those of the poorer classes.

### Bible Stories for Children.

The importance to a Christian nation of definite religious instruction to the child, based upon the Bible and Christ's teaching, has been made plain by the results of its absence, and the substitution for it of "Kultur" and Pagan methods, throughout

* Previous articles appeared on September 23 and 30.

the German Empire, the brutalising effects of which Germany has exhibited to an outraged world in increasing volume since the great war began. On the other hand, the joyous welcome which children extend to the expounding of the Bible and its teachings in the hands of a man or woman who is conscious of his or her spiritual nature and responsibilities, demonstrates the unconscious affinity every child has to its Creator. Many examples of this might be given. Give a child of average intelligence a family Bible which contains pictures, and leave them together for a time. The almost invariable result is that the child comes to its parents, or its teacher, and begs that the pictures may be explained to it, and as an explanation is given, the child asks for more and more, continually reverts to the subject, and shows a desire to have the great Book to look at frequently. As the child gets older, the wish for more and more Bible stories, with an explanation of the meaning of those stories, and of the teaching which naturally flows from Bible illustrations with their explanation, are other striking proofs of the way in which the Bible appeals to it.

At least twenty-five years ago we were lunching with a leading member of the medical profession in Montreal, Canada, it being a family party, with the children present. Long before the meal was finished the children became restless, and at length the mother said, "You may go." They went with a rush, and inquiry revealed the fact that they were keenly anxious not to miss any portion of the Sunday-school class, which they found so attractive and interesting, we were assured, that the children would rather forfeit anything than miss being present there on Sunday. We have had a large experience of children in many places and countries as well as at home, but we have never once seen greater keenness in regard to any matter than those Canadian children displayed in regard to their Sunday-school instruction.

### Ministerial Shortcomings.

The West End of London to-day (1916) is so devoid of evidence of the presence among its population of spiritual leaders and preachers who are conscious of their spiritual nature, with a few notable exceptions, that men and women in large and increasing numbers, in proportion to their knowledge of their spiritual as well as their physical natures and requirements, have experienced a keen sense of spiritual starvation, which has troubled them with an increasing sense of loneliness for the want of a House of God, where His Presence in the sanctuary is recognised and felt by the officiating clergy or ministers. In other words, convention, the habit of perfunctory conduct of the service, the poor selection of the hymns, and the quality and character of the music convey to the quickened sense of the spiritually minded attendant. a feeling of sorrow and emptiness, which has been emphasised and made unbearably oppressive in many places of worship since the war began. Remonstrance, and a prayer for more life in the services, and more direct outspokenness from the pulpit, have produced the explanation that if such a change was to be brought about the congregation would give up their seats. They have grown accustomed to dullness and platitudes, and not even the war has inspired them with the truth that earnest and bright services and direct preaching would prove acceptable, and fill their churches to repletion. In one case, where the music was dull to distraction, the chanting of the Psalms and the chants selected being so heavy as to prove burdensome to the devout, it appeared on investigation that the organist chosen was an East Indian convert, who, in his Mohammedan days, had come to believe that dirge-like and sacred music were synonymous. In this same West End of London we recently had our attention drawn to a church where for some years the spiritual head had abolished the Sunday school. On his retirement, his successor reorganised this school, which has been welcomed by the children as well as their parents, and is making vigorous progress. Some years ago Bishop Neligan, when rector of a West End parish, taught and practised that the Bible lesson should be given in God's House on weekdays instead of in school, with the result that the attendance was inspiriting and quite irrespective of sects. He further had a children's service every Sunday afternoon, which was largely attended and to which many adults made a point of going, for Bishop Neligan is a very able teacher, and the sympathy between him and the children, the clearness of his expositions, and the skill with which he asked questions and utilised the answers, made the Bible a living power for good to everyone present, and carried with it lasting effects.

### Carlyle's Plain Speaking.

Do not these examples enforce Carlyle's declaration to "good Christian people," and demonstrate that where they, or any of them, take heed of the inestimable loan—the child—and employ it in all carefulness, the recompense will be high; and to that recompense may be added the joy and hope of men and women who have become alive to their spirituality, through the blessing of children who have been taught the fear of God and have the love of God in their hearts? May we not hope, for the sake of each individual man and woman in our own nation and the nations of our Allies, that the awakening in each of them of a sense of their spiritual nature may knit together in closer and deeper ties parents and children, and that the real pleasures of life may become paramount, as opposed to the transient pleasures of existence which before the war cramped the horizon and dulled the senses of an ever-increasing number of the population? May we hope that each reader will bear in mind this question, and do his or her utmost to make the answer to it a living force when the moment arrives? In the spiritual sense, how many of the nation, after the war, will recognise their spiritual nature and "become as little children"?

**Next week's article will deal with "Riches and Wealth."**

